# A chemotherapy response classifier based on support vector machines for high-grade serous ovarian carcinoma

**DOI:** 10.18632/oncotarget.6569

**Published:** 2015-12-12

**Authors:** Chao-Yang Sun, Tie-Fen Su, Na Li, Bo Zhou, En-Song Guo, Zong-Yuan Yang, Jing Liao, Dong Ding, Qin Xu, Hao Lu, Li Meng, Shi-Xuan Wang, Jian-Feng Zhou, Hui Xing, Dan-Hui Weng, Ding Ma, Gang Chen

**Affiliations:** ^1^ Department of Obstetrics and Gynaecology, Tongji Hospital, Tongji Medical College, Huazhong University of Science and Technology, Wuhan, Hubei 430030, China; ^2^ Department of Pathology, Tongji Hospital, Tongji Medical College, Huazhong University of Science and Technology, Wuhan, Hubei 430030, China; ^3^ Department of Haematology, Tongji Hospital, Tongji Medical College, Huazhong University of Science and Technology, Wuhan, Hubei 430030, China; ^4^ Department of Obstetrics and Gynecology, Xiangyang Central Hospital, Affiliated Hospital of Hubei University of Arts and Science, Xiangyang, Hubei 441021, China

**Keywords:** ovarian cancer, chemoresistance, support vector machine

## Abstract

Long-term outcome of high-grade serous epithelial ovarian carcinoma (HGSOC) remains poor as a result of recurrence and the emergence of drug resistance. Almost all the patients were given the same platinum-based chemotherapy after debulking surgery even though some of them are naturally resistant to the first-line chemotherapy. No method could verify this part of patients right after the surgery currently. In this study, we used 156 paraffin-embedded high-grade HGSOC specimens for immunohistochemical analysis with 37 immunology markers, and association between the expression levels of these markers and the chemoresponse were evaluated. A support vector machine (SVM)-based HGSOC prognostic classifier was then established, and was validated by a 95-patient independent cohort. The classifier was strongly predictive of chemotherapy resistance, and divided patients into low- and high-risk groups with significant differences progression-free survival (PFS) and overall survival (OS). This classifier may provide a potential way to predict the chemotherapy resistance of HGSOC right after the surgery, and then allow clinicians to make optimal clinical decision for those potentially chemoresistant patients. The potential clinical application of this classifier will benefit those patients with primary drug resistance.

## INTRODUCTION

Epithelial ovarian carcinoma (EOC) is the fifth leading cause of cancer death in women [1]. The 5-year survival rates for late-stage EOC were < 10% between 2004 and 2008 [2]. Long-term outcome remains poor as a result of recurrence and the emergence of drug resistance after original debulking surgery and chemotherapy, especially for the high-grade serous epithelial ovarian carcinoma (HGSOC).

HGSOC is highly heterogeneous. Conventional prognostic features such as patient age, International Federation of Gynecology and Obstetrics (FIGO) stage, histological grade, and initial surgery results are insufficient to capture individual variations in chemoresponse and prognosis. All patients receive the same regimen regardless of their chemoresponse. Therefore, it is important to develop methods that can identify patients who may be resistant to traditional platinum-based chemotherapy and then redirect them to alternative, potentially more efficacious, chemotherapeutic agents (e.g., topotecan) or radiation therapy [3], which may help to improve their overall survivalts.

Molecular prognostic markers could potentially be represented by changes in gene copy number, mRNA and protein expression levels. For example, large-scale RNA expression profiling has been used to screen molecular markers associated with response to chemotherapy and prognosis in ovarian carcinomas [4–7]. However, mRNA expression detection generally requires fresh or frozen tissue that must be microdissected to remove associated non-tumor tissue that may attenuate the gene expression signature of the tumor. Furthermore, mRNA expression was not always correlated with protein levels [8]. In the clinical setting, immunohistochemistry (IHC) remains the most robust and widespread means to evaluate protein abundance in the neoplastic cells or stromal cells, since it only need paraffin-embedded (FFPE) tumor tissues that are routinely prepared in medical practice and are easy to store. Many studies have reported the assessment of single IHC markers for prognosis in ovarian cancer [9–13], but no consistent results have been obtained. We hypothesised that the value of prognostic predictors would be greatly enhanced by using a cluster of specific features, including multiple IHC markers. Support-vector machine (SVM) is a state-of-the-art classification algorithm that can take a small subset of highly discriminating genes to build extremely reliable cancer classifiers. This approach has not yet been applied to HGEOC. In this study, we developed a SVM-based prognostic classifier to predict the chemotherapy response of HGSOC which provides a more accurate measurement of chemotherapy response than is possible through traditional clinical means.

## RESULTS

### Patient characteristics and components of HGSOC-SVM classifier

Table 1 shows the demographic, clinical, and tumor characteristics of the patients in the discovery and validation cohorts. There was no significant difference in clinical or tumor characteristics between the training and validation cohorts. Moreover, we find that only the residual tumor (> 1 cm) is associated with chemotherapy resistance in validation cohort (Supplementary Table 1).

**Table 1:** Clinicopathological characteristics of ovarian cancer patients in training and validation cohorts

Variables	Total*N* = 251	Training *n* = 100 *n* (%)	Validation *n* = 56 *n* (%)	Independent cohort *n* = 95 *n* (%)	*P* value
**Age**					
mean (SD)	50.7 (9.7)	51.3 (9.4)	51.3 (9.3)	49.2 (10.2)	0.171
**Stage**					
IIIC	213	85 (85.0)	44 (78.6)	84 (88.4)	0.264
IV	38	15 (15.0)	12 (21.4)	11 (11.6)	
**Grade**					
moderate	77	30 (30.0)	19 (33.9)	28 (29.5)	0.833
low	174	70 (70.0)	37 (66.1)	67 (70.5)	
**Residual tumor**					
≤ 1 cm	167	63 (63.0)	38 (67.9)	66 (69.5)	0.615
> 1 cm	84	37 (37.0)	18 (32.1)	29 (30.5)	
**Chemotherapy response^#^**					
sensitive	151	57 (57.0)	34 (60.7)	60 (63.2)	0.677
resistant	100	43 (43.0)	22 (39.3)	35 (36.8)	

Chemotherapy response^#^ was defined as relapse or progression within 6 months or later than 6 months from the last platinum-based chemotherapy, respectively.

**Figure 1:**
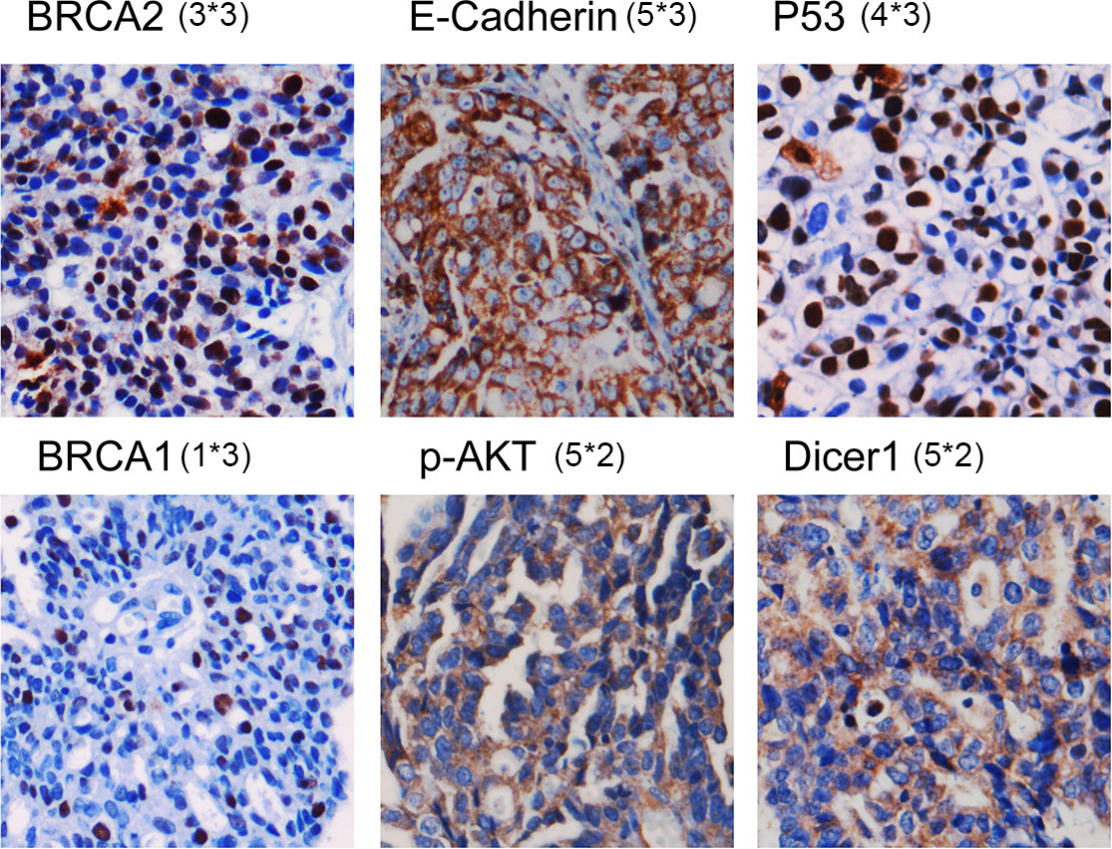
Representative IHC staining for these six SVM-RFE–selected markers in HGSOC tumor tissues The scores of staining intensity and pattern of staining of these representative images were showed in a format as A*B. A refers to the scores of staining intensity, while B represents the scores of pattern of staining. And the aggregate scores will be the scores of tumor-cell staining multiplied by the score of staining intensity.

On the basis of SVM-RFE analysis of the 100 cases of the training set, the final HGSOC-SVM classifier integrated one clinicopathologic feature (optimal debunking surgery) and expression of 6 proteins, BRCA2, E-Cadherin, P53, BRCA1, p-AKT, Dicer1, as critical factors. Representative IHC staining for these six SVM-RFE selected markers in HGSOC tumor tissues is shown in Figure 1. Raw data was provided as the Supplementary Table 2.

### HGSOC-SVM classifier and chemotherapy resistance of HGSOC

**Figure 2:**
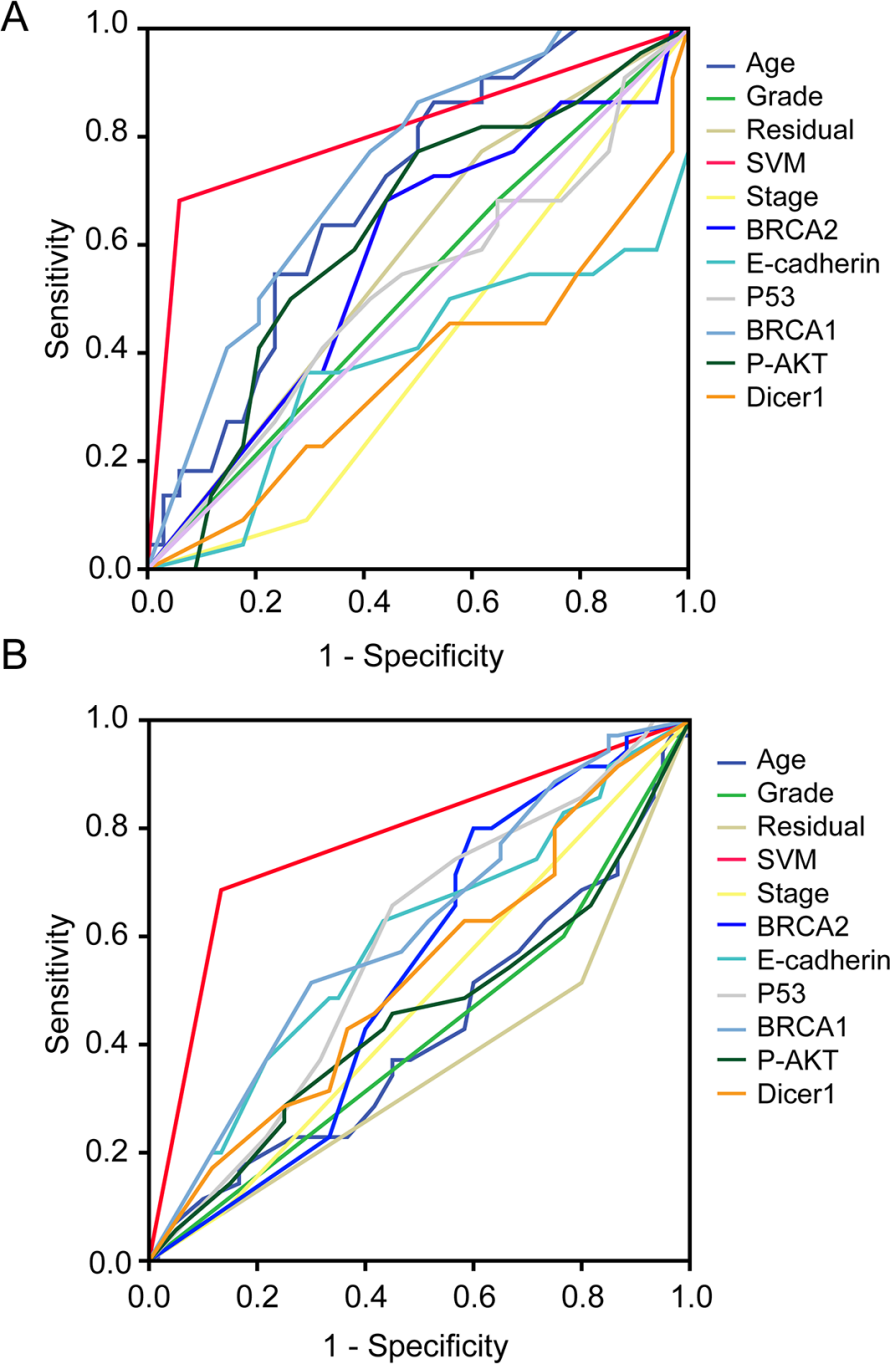
Receiver operating characteristic (ROC) curves for traditional clinicopathological prognostic factors, including age, and clinical stage, grade, residual tumor volume, as well as each 6 selected molecular marker and the HGSOC-SVM classifier in both of testing and validation cohort (**A**) ROC curves for 56 testing patients in discovery cohort; (**B**) ROC curves for 95 patients in validation cohort.

ROC curves for traditional clinicopathological prognostic factors, including age, and clinical stage, grade, residual tumor volume as well as each individual molecular marker and the HGSOC-SVM classifier in both the testing and validation cohorts sets, are illustrated in Figure 2. In the testing cohort (*n* = 56), the HGSOC-SVM classifier (AUC = 0.802) outperformed all the other individual prognostic factors (Figure 2A). The HGSOC-SVM classifier was strongly predictive of chemotherapy resistance (overall accuracy, 83.9%; sensitivity, 94.1%; specificity, 68.2%). These prognostic associations were also observed in the independent validation cohort (*n* = 95) (AUC = 0.776) (Figure 2B) including prediction of chemotherapy resistance (overall accuracy, 80.0%; sensitivity, 86.7%; specificity, 68.6%).

In univariate logistic analysis based on the testing cohort (Supplementary Table 3), the high-risk group based on the HGSOC-SVM classifier was highly associated with chemotherapy resistance (OR = 34.3, 95% CI: 6.35 to 185.24, *P* < .001). By contrast, there was no significant difference in chemotherapy resistance by age, histological grade, clinical stage, or optimal surgery. Similarly, in the validation cohort, high-risk group according to the HGSOC-SVM classifier was also the most important predictive factor for chemotherapy resistance (OR = 14.18, 95% CI: 5.05 to 39.77, *P* < .001) (Supplementary Table 3).

### HGSOC-SVM classifier and HGSOC OS and PFS

**Figure 3:**
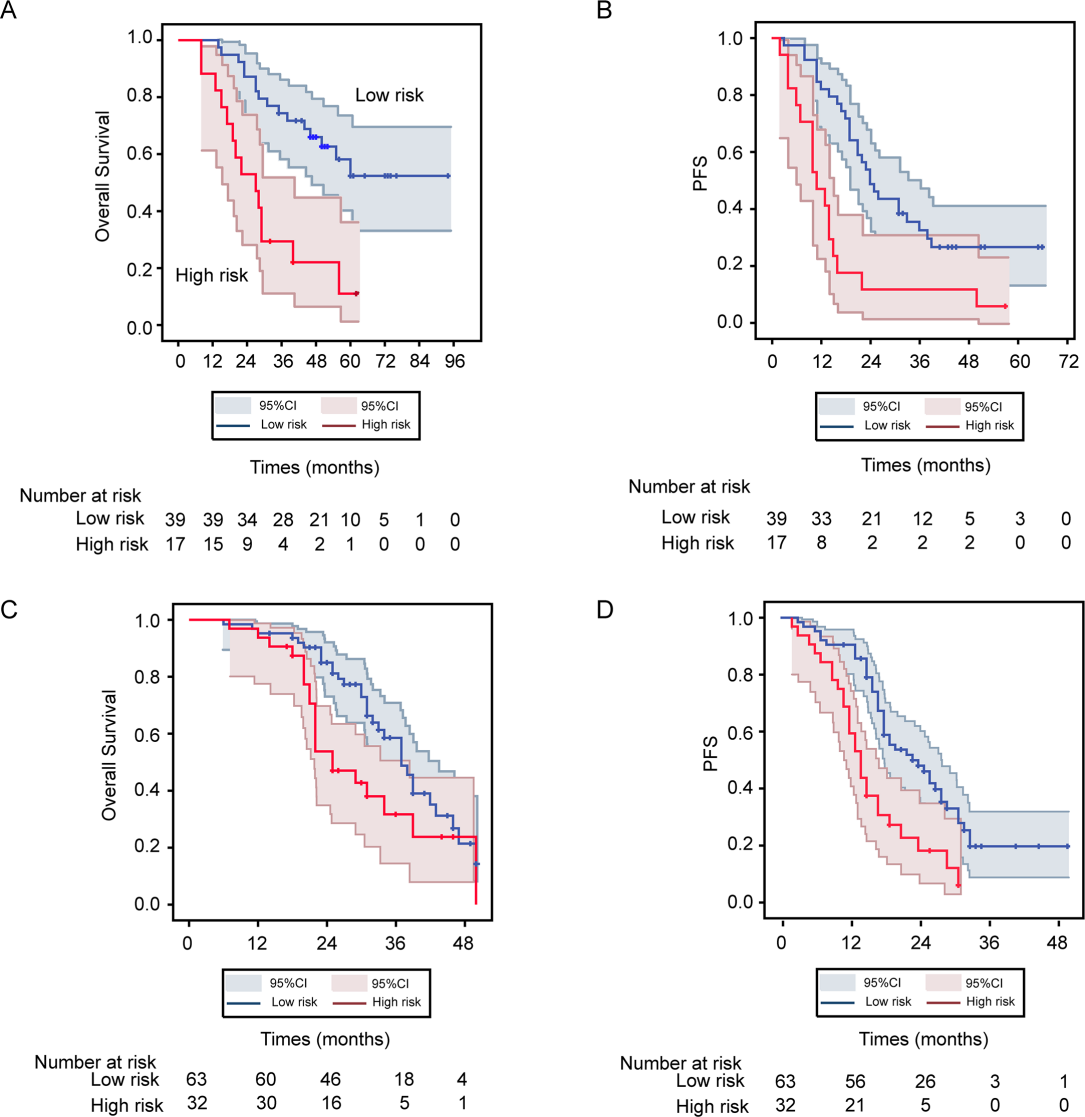
Kaplan-Meier OS and PFS estimate for low- and high-risk patients with HGSOC as defined by HGSOC-SVM classifier from both training and validation cohorts (**A**) Kaplan-Meier OS curves for 56 testing patients in discovery cohort; (**B**) Kaplan-Meier OS curves for 95 patients in validation cohort; (**C**) Kaplan-Meier PFS curves for 56 testing patients in discovery cohort; (**D**) Kaplan-Meier PFS curves for 95 patients in validation cohort. Log-rank test used to calculate *P* values.

In the 56 testing patients, HGSOC-SVM classifier defined 39 patients as low risk and 17 patients as high risk. OS differed significantly between low- and high-risk patients (median OS: 50.0 months, 95%CI: 41.8 to 53.5 months vs. 27 months, 95%CI: 19.5 to 35.2 months, *P* < .001) (Figure 3A). In the validation cohort of 95 patients, the HGSOC-SVM classifier was used to define 63 patients as low risk and 32 patients as high risk. Again, the OS differed strikingly between low- and high-risk patients (*P* = 0.017) (Figure 3B). PFS differed significantly between low- and high-risk patients in both the 56 testing patients (median PFS: 24.0 months, 95% CI: 19.1 to 28.9 months vs. median PFS: 11 months, 95% CI: 6.2 to 15.8 months, *P* < .001) (Figure 3C) and 95 patients of the independent validation cohort (median PFS: 23.0 months, 95% CI: 16.4 to 29.6 months vs. median PFS: 14 months, 95% CI: 11.8 to 16.2 months, *P* < .001) (Figure 3D). Univariate associations of the HGSOC-SVM classifier, clinicopathological parameters, and expression of each of the 6 immunological markers with OS and PFS in the 56 testing patients and in the 95 patients from validation cohort are shown in Tables 2 and 3. Only the HGSOC-SVM classifier showed significant association with OS in both groups (HR = 3.90, 95% CI: 1.87 to 8.16, *P* < .001; and HR = 1.92, 95% CI: 1.10 to 3.34, *P* = 0.022, respectively). Similarly, only the HGSOC-SVM classifier had consistent statistically significant prognostic value for PFS in both groups (HR = 2.92, 95% CI: 1.55 to 5.50, *P* < .001 and HR = 2.38, 95% CI: 1.45 to 3.91, *P* < .001, respectively).

**Table 2:** Association between SVM model and clinicopathological characteristics of ovarian cancer patients and OS in testing patients of discovery cohort and in validation cohort

Variables	Testing patients		Validation cohort	
	HR (95% CI)	*P* value	HR (95% CI)	*P* value
SVM (high vs. low)	3.90 (1.87–8.16)	< 0.001*	1.92 (1.10–3.34)	0.022*
Residual tumor (> 1 cm vs. ≤ 1 cm)	1.47 (0.65–3.31)	0.351	0.60 (0.34–1.06)	0.077
Grade (low vs. moderate)	0.53 (0.26–1.08)	0.079	0.81 (0.45–1.46)	0.494
Stage (III C vs. IV)	0.56 (0.22–1.48)	0.244	0.53 (0.19–1.53)	0.242
Age (> 55 y vs. ≤ 55 y)	1.71 (0.82–3.58)	0.152	0.68 (0.39–1.17)	0.164
BRCA2	1.75 (0.83–3.67)	0.142	1.72 (0.91–3.22)	0.093
E-Cadherin	0.68 (0.29–1.59)	0.373	1.14 (0.66–1.95)	0.642
P53	1.51 (0.73–3.12)	0.263	1.41 (0.82–2.44)	0.215
BRCA1	1.35 (0.61–2.95)	0.458	1.32 (0.76–2.30)	0.328
p-AKT	1.60 (0.78–3.30)	0.199	1.10 (0.51–2.38)	0.799
DICER1	0.65 (0.31–1.33)	0.234	1.24 (0.72–2.15)	0.434

HR = Hazord ratio; CI = confidence interval. *P < 0.05

**Table 3:** Association between SVM model and clinicopathological characteristics of ovarian cancer patients and PFS in testing patients of discovery cohort and in validation cohort

Variables	Testing patients (*n* = 56)		Validation cohort (*n* = 95)	
	HR (95% CI)	*P* value	HR (95% CI)	*P* value
SVM (high vs. low)	2.92 (1.55–5.50)	0.001*	2.38 (1.45–3.91)	0.001*
Residual tumor (> 1 cm vs.≤ 1 cm)	1.41 (0.74–2.70)	0.299	0.45 (0.27–0.76)	0.003*
Grade (low vs. moderate)	0.91 (0.49–1.69)	0.774	0.78 (−47–1.30)	0.336
Stage (III C vs. IV)	0.76 (0.36–1.58)	0.464	0.70 (0.30–1.63)	0.411
Age (> 50 y vs. ≤ 50 y)	1.85 (1.01–3.39)	0.047	0.82 (0.51–1.31)	0.398
BRCA2	1.42 (0.78–2.59)	0.256	1.23 (0.74–2.05)	0.418
E-Cadherin	0.39 (0.20–0.75)	0.005*	1.32 (0.82–2.11)	0.251
P53	1.51 (0.83–2.76)	0.175	1.43 (0.89–2.29)	0.142
BRCA1	2.61 (1.31–5.18)	0.006*	1.23 (0.76–1.99)	0.407
p-AKT	1.59 (0.87–2.90)	0.133	0.72 (0.42–1.25)	0.239
DICER1	0.92 (0.51–1.68)	0.797	0.99 (0.61–1.60)	0.961

OR = Hazord ratio; CI = confidence interval. *P < 0.05

## DISCUSSION

Identification of patents with likely chemoresistant before the commencement chemotherapy would greatly aid clinical management. Traditionally, clinical factors such as age and tumor grade have been used to assess prognosis, however they have poor predictive power [14, 15]. Immunological biomarkers may have superior prediction capacity. Many studies have reported the value of single prognostic immunomarkers in ovarian cancer, but no consistent results have been obtained [9–13].

To improve the prognostic predictive value of individual genes, supervised methods, such as decision trees, artificial neural network, could be used to combine independently informative markers to improve predict values [16–18]. SVM is one of the most classic supervised learning algorithms, useful for recognizing subtle patterns in complex datasets. The algorithm performs discriminative classification, learning by example to predict the classifications of previously unclassified data. Compared with other machine learning algorithms, SVM is well suited to managing high-dimensional data in limited numbers of training samples. Through the recursive feature elimination (RFE) technique, SVM models are also effective for discovering informative features or attributes from a large number of candidate features. In SVM-RFE analysis, the SVM framework was used to evaluate all the markers collectively with the least important marker first identified by the SVM subsequently eliminated. This process is repeated until only one marker is left. SVM-RFE is especially useful to determine the independent contribution of each marker for model performance. Many studies have reported that the combination of gene expression and clinical data leads to better classifiers for predicting a patients' outcome. Thus, SVM model is appropriate for integrate clinicopathologic features with predominant genes to predict the outcome of patients. In this study, our HGSOC-SVM classifier integrated optimal surgery and expression of six proteins (BRCA2, E-Cadherin, P53, BRCA1, p-AKT and Dicer1). The genes identified in this SVM classifier are in commonly deregulated pathways in ovarian cancer [14, 19, 20]. BRCA1/BRCA2, P53, the EMT phenotype (particularly E-Cadherin expression) and Dicer1 expression have independently been reported as a prognostic factors for chemotherapy resistance and survival of women with ovarian carcinoma [19–24]. We also have reported that higher BRCA1 expression is associated with chemosensitivity in ovarian cancer patients [25], and ovarian cancer patients with BRCA dysfunction tend to have a better outcome [11]. What's more, these markers also hold considerable promise as therapeutic targets. Agents targeting p53, p-AKT are currently under investigation in clinical trials. Many studies have shown that tumors with BRCA1/BRCA2 dysfunction are highly responsive to PARP inhibitors [26]. Our results indicate that when each of these molecules individually in HGSOC was weakly associated with chemoresistance, PFS and OS, the HGSOC-SVM classifier was substantially stronger than any single component. Thus, HGSOC-SVM classifier was able to select the most informative factors that contributed independently and collectively to the prediction of HGSOC prognosis.

The study has a few limitations. Although we validated our findings in an independent cohort, more samples from multicenter will be needed to strengthen our results in the future, which may increase sensitivity and specificity. Additionally, we chose candidate proteins based on published studies, not whole-genome analyses, and thus we cannot exclude the possibility of better models comprising other molecular biomarkers. Finally, our results are based on retrospective data, we need more prospective validation to gain the possibility that this method could help clinical decision making and improving outcome for those potentially chemoresistant patients.

In summary, we have developed an SVM-based prognostic method to predict chemotherapy response in ovarian cancer patients; this method has high sensitivity and specificity, with high positive and negative predictive value. This HGSOC-SVM classifier predicts the chemoresponse, PFS and OS of patients better than any other clinical parameters. Although further validation is necessary, this prognostic strategy may allow clinicians to select the most appropriate therapies, apart from the standard paclitaxel and cisplatin approach, for individual ovarian cancer patients in advance, especially potentially chemoresistant patients.

## METHODS

### Patient selection

With the approval and support of the Ethics/Institutional Review Board of Tongji Hospital and Hubei Cancer Hospital (Wuhan, Hubei Province, China), HGSOC patients with FIGO stage III_C_ or IV were identified. All patients were primary, biopsy-confirmed HGSOC who underwent debulking surgery and subsequent platinum/taxane-based chemotherapy. Informed consent was obtained from all patients. We enrolled two independent sets of HGSOC patients, including a discovery cohort of 156 patients diagnosed between October 2001 and July 2009 at Tongji hospital and a validation cohort of 95 patients with HGSOC diagnosed between October 2009 and December 2012 at Tongji Hospital and Hubei Cancer Hospital. Clinical features are listed in Table 1.

Follow-up information was updated in Jun 2014 through the patients' medical records and telephone based follow-up review. Chemotherapy resistance or sensitivity was defined as tumor relapse/progression within 6 months or 6 months after completion of prior platinum-based chemotherapy, respectively. Primary therapy response was defined as response evaluation criteria in solid tumors (RECIST). Progression-free survival (PFS) was calculated from time of surgery to time of progression or recurrence. Optimal debulking was defined as when residual tumors were ≤ 1 cm.

### Tissue microarray construction and IHC

After review of HE-stained sections, three 1.5 mm cores were identified from the most representative areas of tumor tissue and re-embedded into tissue microarray blocks with a Manual Tissue Microarrayer (Beecher Instruments, Sun Prairie, WI, USA). Array blocks were sectioned to produce 4 μm sections.

Candidate protein markers used to build new prognostic classifiers in this study were selected from previous literatures, and involved in different aspects of chemotherapy resistance of HGSOC, including ABC transporter (LRP, MRP1, MRP2, ABCA1, SLC31A1), microtubule assembly (TUBB3), DNA repair (ERCC1, BRCA1, Rad51, BRCA2, PARP1, 53BP1), cell cycle (Cyclin D1, CDK1, Ki67, Cyclin E1, P21WAF1, P27kip1), apoptosis (BAX, BCL-2, HSP27), epithelial-mesenchymal transition (EMT) (E-cadherin, Vimentin, transforming growth factor-β [TGF-β1], β-catenin), proto-oncogenes (MYC, FAK, HER2, EGFR, p-AKT), cancer stem cell marker (ALDH1, OCT4), ROS detoxification (SOD1, GSTP1), MicroRNA processing (Dicer1) and tumor suppressor (P53, PTEN) genes. IHC staining was performed according to standard procedures [27]. The information of these biomarkers is summarized in Supplementary Table 4. Appropriately selected positive-control samples were used according to the manufacturers' instructions and included in each batch. For a negative-control samples were processed as per the standard protocol but without the primary antibodies from the dilution buffer.

### IHC scoring

Tissue samples were scored manually using methods previously described [17]. Briefly, the aggregate score is the average of the score of tumor-cell staining multiplied by the score of staining intensity. Tumor-cell staining was assigned a score using a semi-quantitative five-category grading system: 0, no tumor-cell staining; 1, 1–10% tumor-cell staining; 2, 11–25% tumor-cell staining; 3, 26–50% tumor-cell staining; 4, 51–75% tumor-cell staining; and 5, > 75% tumor-cell staining. Staining intensity was assigned a score using a semi-quantitative four-category grading system: 0, no staining; 1, weak staining; 2, moderate staining; and 3, strong staining. Every core was assessed individually and the mean of the three readings was calculated for every case. The pattern of staining (cytoplasmic, membranous, nuclear) was also described in each case. Two trained histopathologists (Dr Su and Dr Li) blinded to clinical data scored all cases and a concordant score was obtained for 85.2% of the cases. A consensus score was recorded for the 14.8% of cases with a discordant score.

### Introduction of SVM and details of experiments

SVM was used to predict whether a patient would have chemotherapy-resistant HGSOC based on clinicopathological features and immunomarkers. We performed a set of experiments in the discovery cohort of 156 HGSOC patients. And this cohort was further randomly subdivided into 100 patients for SVM training, and 56 for testing. To make an accurate assessment of the model, we re-enrolled an independent cohort of 95 patients in accordance with the modeling standards. These patients were input into the model as a double-blind test set to evaluate and calculate the sensitivity and specificity of the model. (Supplementary Figure 1)

Before training, all continuous data were preprocessed by standardizing to zero mean and unit variance. The same offset and scaling were applied to the test data. We adopted the SVM-recursive-feature-elimination (SVM-RFE) algorithm for feature selection. The radial basis function kernel was used, since our classification problem was nonlinear. During the training phase, 10-fold cross validation was used to determine the optimal values of the kernel parameter *a* and the regularization parameter C with a 10 × 10 grid search in the region −10 < log_2_ *a* < 10 and −10 < log_2_ *C* < 10, and step size of log_2_1. This algorithm is performed 40 times, and each time, one feature subset is excluded. During the training, we evaluated the performance of SVM using 10-fold cross validation error. After the feature subset with the best 10-fold cross validation performance was selected, we predicted the labels of tested and validation samples and recorded the actual performance of the trained SVM models. The programs were coded with Matlab software (MathWorks, Natick, MA, USA).

### Statistical analysis

Differences in patient characteristics between different groups were tested with Pearson *χ*^2^ test. To identify the association between all features with chemotherapy resistance, univariate Logistic regression was performed. Survival curves were estimated by the Kaplan-Meier method. Differences between survival curves were compared by the log-rank test. Univariate analyses of prognostic factors were performed with Cox proportional hazards regression modeling. In Logistic and Cox proportional hazards regression analysis, all immunological features were dichotomized according to cutoff values based on receiver operating characteristic (ROC) curve analysis. A significant difference was defined as a *P* value of ≤ .05 from a two-tailed test. All statistical analyses were performed with SPSS 16.0 for Windows (SPSS, Chicago, IL, USA).

## SUPPLEMENTARY FIGURES AND TABLES




