# Supplementary material for: A chemotherapy response classifier based on support vector machines for high-grade serous ovarian carcinoma
Source: Oncotarget. 2015 Dec 12;7(3):3245–54. doi: 10.18632/oncotarget.6569 (PMC4823103; doi:10.18632/oncotarget.6569)
Supplement: Supplementary file 1 [file oncotarget-07-3245-s001.pdf]

A chemotherapy response classifier based on support vector machines for high-grade serous ovarian carcinoma

Supplementary Materials

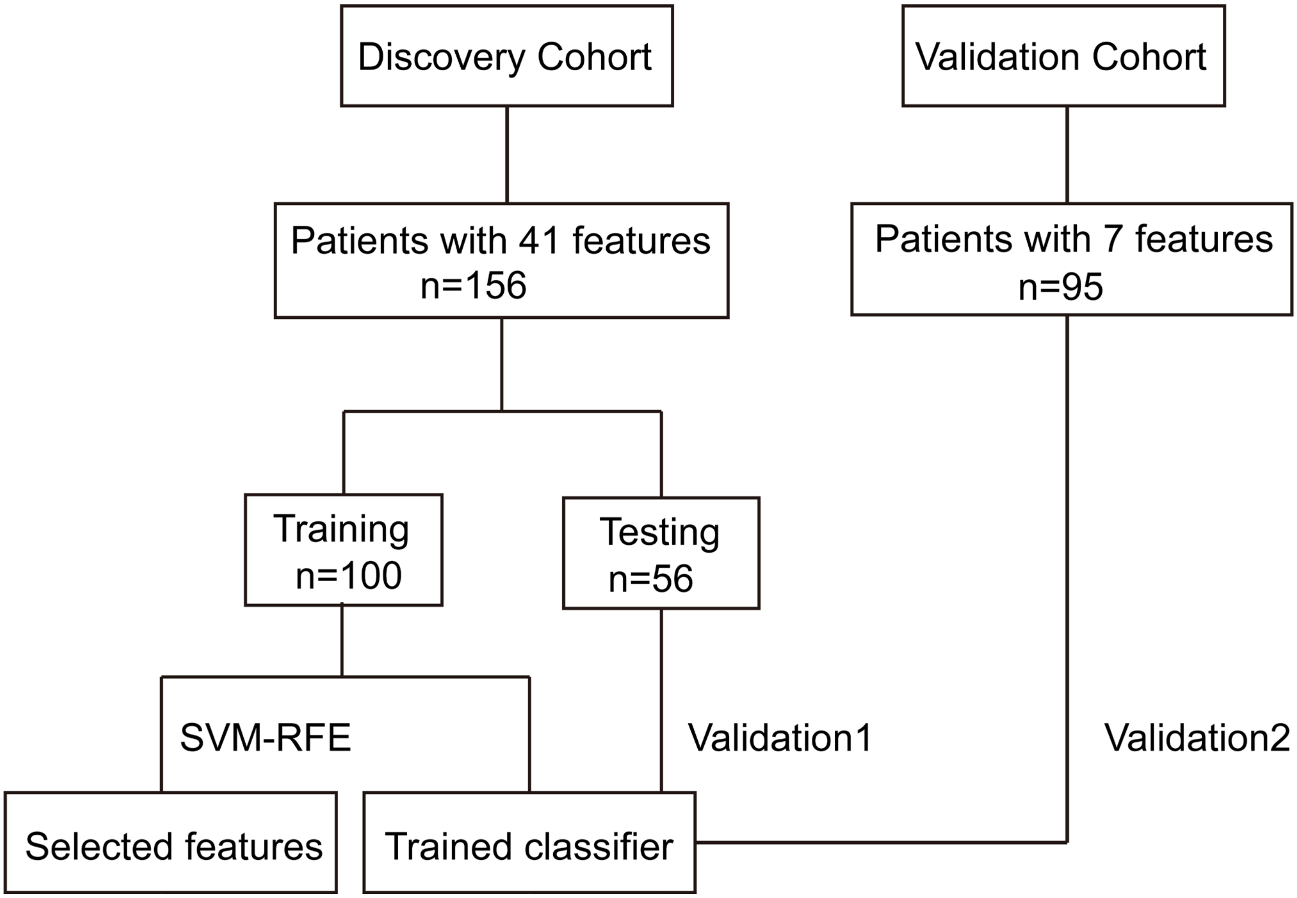

Supplementary Figure S1: Flow chart for the support vector machine (SVM) models used to derive the SVM classifier for this study.

Supplementary Table 1: Clinicopathological characteristics of ovarian cancer patients in Discovery and Validation Cohorts between sensitive and resistant patients

Variables	Discovery dataset			Validation dataset		
	Sensitive *n* (%)	Resistant *n* (%)	*P* value	Sensitive *n* (%)	Resistant *n* (%)	*P* value *n* (%)
**Age**						
mean (SD)	51.2 (10.4)	49.7 (9.9)	0.874	49.8 (11.3)	50.2 (9.8)	0.789
**Stage**						
IIIC	72 (79.1)	57 (87.7)	0.200	52 (86.7)	32 (91.4)	0.741
IV	19 (20.9)	8 (12.3)		8 (13.3)	3 (8.6)	
**Grade**						
moderate	27 (29.7)	22 (33.8)	0.603	14 (23.3)	14 (40.0)	0.105
low	64 (70.3)	43 (66.2)		46 (76.7)	21 (60.0)	
**Residual tumor**						
≤1cm	55 (60.4)	46 (70.8)	0.234	48 (80.0)	18 (51.4)	0.005
>1cm	36 (39.6)	19 (29.2)		12 (20.0)	17 (48.6)	
**Platinum**						
Cisplatin	34 (37.4)	19 (29.2)	0.309	18 (30.0)	16 (45.7)	0.183
Carboplatin	57(62.6)	46(70.8)		42(70.0)	19(54.3)	
**Taxel**						
Paclitaxel	84 (92.3)	61 (93.8)	0.763	55 (91.7)	33 (94.3)	1.000
Docetaxel	7 (7.7)	4 (6.2)		5 (8.3)	2 (5.7)	

Supplementary Table 3: Association between clinicopathological characteristics and immunological markers with chemotherapy resistance in testing patients of discovery cohort and independent validation cohort

Variance	Testing cohort (*n* = 56)			Independent Validation cohort (*n* = 95)		
	**OR**	**95% CI**	***P***	**OR**	**95% CI**	***P***
SVM (high vs. low)	34.29	6.35, 185.24	**< 0.001***	14.18	5.05, 39.77	**< 0.001***
Age (> 50 y vs. ≤ 50 y)	3.06	0.99, 9.45	0.052	0.53	0.23, 1.25	0.147
Stage (IV vs. III C)	0.24	0.05, 1.23	0.086	0.61	0.15, 2.47	0.487
Residual Tumor (> 1 cm vs. ≤ 1 cm)	2.11	0.63, 7.08	0.229	0.26	0.11, 0.66	**0.004***
Grade (low vs. moderate)	1.17	0.37, 3.66	0.789	0.46	0.19, 1.13	0.089
BRCA2	2.71	0.88, 8.35	0.082	2.67	1.01, 7.07	**0.049***
E-Cadherin	0.19	0.05, 0.74	**0.016***	2.21	0.94, 5.20	0.069
P53	1.35	0.46, 3.96	0.585	2.34	0.99, 5.56	0.053
BRCA1	6.33	1.58, 25.45	**0.009***	2.47	1.04, 5.85	**0.040***
p-AKT	2.51	0.83, 7.57	0.103	0.43	0.16, 1.12	0.084
DICER1	0.66	0.22, 1.93	0.447	1.29	0.55, 3.03	0.551

All immunological features were dichotomized according to cutoff values based on receiver operating characteristic (ROC) curve analysis.
OR = odds ratio; CI = confidence interval. *$P < 0.05$

Supplementary Table S4: List of immunomarkers used for immunohistochemical staining of high-grade serous ovarian carcinoma (HGSOC)

Gene	Clone	Catalog	Dilution	Antigen Retrieval	Positive Signal	Source
BRCA2	Mouse monoclonal	MAB2476	1:30	EDTA	Nuclear	R&D
E-Cadherin	24E10	#3195	1:200	Citric acid high pressure	Membranous	CST
P53	DO-7	M7001	1:400	EDTA	Nuclear	DAKO
BRCA1	MS110	ab16780	1:50	Citric acid high pressure	Nuclear	Abcam
p-AKT	D9E	#4060	1:50	Citric acid high pressure	Cytoplasmic	CST
DICER1	13D6	ab14601	1:100	Citric acid high pressure	Cytoplasmic	Abcam
HER2	Rabbit polyclonal	ab2428	1:100	Citric acid high pressure	Membranous	Abcam
ALDH1	44/ALDH	611195	1:100	Citric acid high pressure	Cytoplasmic	BD
BCL-2	N-19	sc-492	1:50	Citric acid high pressure	Cytoplasmic	Santa Cruz
BAX	B-9	sc-7480	1:50	Citric acid high pressure	Cytoplasmic	Santa Cruz
CDK1	E161	ab32384	1:100	Citric acid high pressure	Cytoplasmic	Epitomics
EGFR	EP38Y	ab52894	1:200	Citric acid high pressure	Membranous	Epitomics
ERCC1	8F1	ab2356	1:50	Citric acid high pressure	Nuclear	Abcam
MYC	9E10	sc-40	1:100	Citric acid high pressure	Cytoplasmic	Santa Cruz
FAK	Rabbit polyclonal	12636-1-AP	1:100	Citric acid high pressure	Cytoplasmic	Proteintech
GSTP1	Rabbit polyclonal	15902-1-AP	1:100	Citric acid high pressure	Cytoplasmic	Proteintech
HSP27	G31	#2402	1:100	Citric acid high pressure	Cytoplasmic	CST
P27 kip1	C-19	sc-528	1:100	Citric acid high pressure	Cytoplasmic	Santa Cruz
KI67	SP6	2746-1	1:300	Citric acid high pressure	Nuclear	Epitomics
LRP	Rabbit polyclonal	16478-1-AP	1:100	Citric acid high pressure	Membranous	Proteintech
MRP1	QCRL-1	sc-18835	1:50	Citric acid high pressure	Membranous	Santa Cruz
MRP2	Rabbit polyclonal	24893-1-AP	1:100	Citric acid high pressure	Cytoplasmic	Proteintech
OCT4	C-10	sc-5279	1:50	Citric acid high pressure	Nuclear	Santa Cruz
P21 WAF1	12D1	#2947	1:100	Citric acid high pressure	Nuclear	CST
Cyclin D1	H-295	sc-753	1:50	Citric acid high pressure	Nuclear	Santa Cruz
SOD1	Rabbit polyclonal	10269-1-AP	1:100	Citric acid high pressure	Cytoplasmic	Proteintech
PARP1	4C10-5	556494	1:200	Citric acid high pressure	Cytoplasmic	BD biosciences
SLC31A1	Rabbit polyclonal	ab133385	1:80	Citric acid high pressure	Membranous	Abcam
TGFB1	Rabbit polyclonal	18978-1-AP	1:50	Citric acid high pressure	Cytoplasmic	Proteintech

Cyclin E1	C-19	sc-198	1:100	Citric acid high pressure	Nuclear	Santa Cruz
TUBB3	Rabbit polyclonal	10094-1-AP	1:100	Citric acid high pressure	Cytoplasmic	Proteintech
Vimentin	EPR3776	2707-1	1:400	Citric acid high pressure	Cytoplasmic	Epitomics
53BP1	Rabbit polyclonal	ab36823	1:1000	Citric acid high pressure	Nuclear	Abcam
PTEN	D4.3	#9188	1:100	Citric acid high pressure	Cytoplasmic	CST
RAD51	EPR4030	5181-1	1:200	Citric acid high pressure	Nuclear	Epitomics
ABCA1	AB.H10	ab18180	1:200	Citric acid high pressure	Membranous	Abcam
β-Catenin	C-18	sc-1496	1:50	Citric acid high pressure	Cytoplasmic	Santa Cruz

CST: Cell Signaling Technology
